# Edible Flowers as a Source of Dietary Fibre (Total, Insoluble and Soluble) as a Potential Athlete’s Dietary Supplement

**DOI:** 10.3390/nu14122470

**Published:** 2022-06-14

**Authors:** Karolina Jakubczyk, Klaudia Koprowska, Aleksandra Gottschling, Katarzyna Janda-Milczarek

**Affiliations:** Department of Human Nutrition and Metabolomics, Pomeranian Medical University in Szczecin, 24 Broniewskiego Street, 71-460 Szczecin, Poland; klaudia1012@interia.pl (K.K.); olagottschling@o2.pl (A.G.); katarzyna.janda.milczarek@pum.edu.pl (K.J.-M.)

**Keywords:** edible flowers, fibre, insoluble fibre, soluble fibre, diet-related diseases, athletes

## Abstract

Edible flowers have been gaining popularity among researchers, nutritionists and chefs all around the world. Nowadays, flowers are used to make food look and/or taste better; however, they are also a very good source of valuable nutrients (antioxidants, vitamins, proteins, fats, carbohydrates, macro and microelements). The aim of our study was to determine the content of dietary fibre and total protein in selected edible flowers; we also compared the nutritional content of petals, differentiating between the representatives of the *Oleaceae* and *Asteraceae* families, as well as herbaceous vs. woody plants. The study material consisted of petals of 12 edible flower species (*Magnolia* × *soulangeana*, *Sambucus nigra* L., *Syringa vulgaris* L. (white and violet flowers), *Robinia pseudoacacia*, *Forsythia* × *intermedia*, *Cichorium intybus* L., *Bellis perennis*, *Tussilago farfara* L., *Taraxacum officinale* F.H. Wiggers coll., *Centaurea cyanus* L., *Calendula officinalis*). Dietary fibre content was determined by the enzymatic-gravimetric method and ranged from 13.22 (*Magnolia* × *soulangeana*) to 62.33 (*Calendula officinalis* L.) g/100 g. For insoluble dietary fibre (IDF), the values ranged from 8.69 (*Magnolia* × *soulangeana*) to 57.54 (*Calendula officinalis* L.) g/100 g, and the content of soluble dietary fibre (SDF) was between 1.35 *(Syringa vulgaris* L.-white flowers) and 7.46 (*Centaurea cyanus* L) g/100 g. Flowers were also shown to be a good, though underappreciated, source of plant protein, with content ranging from 8.70 (*Calendula officinalis* L.) to 21.61 (*Magnolia* × *soulangeana*) g/100 g dry matter (Kjeldahl method). Considerable amounts of protein were found in the flowers of the olive family (*Oleaceae*) and woody plants, which can enrich the daily diet, especially vegan and vegetarian. Edible flowers of the *Asteraceae* family, especially the herbaceous representatives, contained high levels of both total dietary fibre and its insoluble fraction; therefore, they can be a rich source of these nutrients in the daily diet of athletes, which would perform a prebiotic function for gut bacteria.

## 1. Introduction

The diet of active people should take care of the proper level of all nutrients, including dietary fibre and protein. Low content of nutrients may reduce performance, prolonged recovery time after physical activity, the possibility of more injuries, and the feeling of fatigue. Additionally, the post-workout meal should contain protein and carbohydrates [1,2]. Research conducted among 610 athletes (391 men and 219 women) confirmed that these people displayed inappropriate eating behaviour. There was an inadequate intake of fruit (59.89%), whole grains (59.90%), vegetables (53.62%) and dairy products (52.09%), which may result in deficiencies in dietary fibre and protein. Compared to women, men to a greater extent did not include raw vegetables (*p* < 0.001) and whole grains (*p* < 0.05) in their daily diet [2]. In addition, physically active people often use dietary supplementation to make up for deficiencies and obtain better sports results [3].

Dietary fibre has long been recognised as an essential component of a healthy diet, with a recommended daily intake of 25–35 g. A high fibre intake has been associated with a reduced risk of several health conditions, including cardiovascular disease, diabetes, hypertension, obesity and gastrointestinal disorders [4]. Additionally, fibre improves bowel function, it is fermentable by intestinal microflora, and lowers blood glucose and cholesterol levels [5].

The physiological effects of dietary fibre can be attributed to its chemical and physical properties such as viscosity, degradability, molecular weight, particle size, cation exchange properties, and water holding capacity [5,6]. The functions of individual fibres vary, and therefore a varied diet is recommended that can provide fibre from different sources [5]. Different fractions and amounts of dietary fibre in a meal can affect the gastric emptying rate, bowel transit time, and nutrient absorption. When it reaches the large intestine, dietary fibre is at least partially fermented, interacting with the intestinal microbiome [7,8,9].

There is a close relationship between the prevalence of chronic diseases, the health status of the population, and the consumption of fibre-rich cereal products. In addition, fibre is a very important nutrient in an athlete’s diet. Unfortunately, it seems that a large percentage of the population has difficulty achieving even the minimum recommended intake, also in athletes [10,11]; it is therefore important to incorporate high-fibre and unprocessed foods that will be appealing to consumers.

Increasingly, athletes or exercisers decide to eat a plant-based diet, which may be associated with deepening deficiencies; moreover, researchers and nutritionists suggest that with proper meal planning, an athlete can meet all of his nutritional needs with plant-based foods without sacrificing physical performance [12,13]. Edible freeze-dried flowers can help to supplement protein or dietary fibre, in addition, they can be easily added to a meal, e.g., a cocktail or yoghurt consumed after training.

Recently, edible flowers have seen a significant rise in popularity. In China and Japan, they have been consumed for thousands of years. Over time, they gained a place on the table in other cultures: in Europe, notably Victorian England, India and the Middle East. New food processing technologies, as well as new logistics methods, enabling rapid distribution of preserved food products, have made possible their broader use [14,15]. The wide variety of species, different flowering times and different organoleptic properties contribute to the great potential and applicability of flowers in many industries. In the food industry and gastronomy, they are used as garnishing as well as an integral part of the meal [14]; they can be used fresh (in salads, cocktails, and even meat dishes), dried (as infusions, dessert garnishing, e.g., dried rose or cornflower petals), powdered, crystallised or as foam (molecular gastronomy) [16,17]. Chefs around the world reach beyond the ordinary when coming up with new recipes. Floral ingredients enhance visual appeal, add a fresh, delicate aroma to dishes and they can also be used as natural colourants; some can be used in food as natural preservatives, flavouring or colouring agents [18]. 

Edible flowers contain bioactive compounds, which are secondary metabolites formed under the influence of environmental stressors, such as temperature, water deficit, excessive UV radiation or pest infestation [19]; these compounds include primarily polyphenols, which exhibit a broad spectrum of biological activities. Edible flowers are also a source of vitamins, essential oils and antioxidants, especially when consumed fresh or minimally processed, e.g., freeze-dried [20]. Following this trend, the biological properties of edible flowers have been linked to reduced severity of ulcerative colitis [21], antihyperglycemic and anticholinergic effects [22], protection against oxidative effects in erythrocytes [23], and even anticancer activity [24]. Additionally, they have been shown to have anti-inflammatory, antimicrobial and gastroprotective properties [25,26]. Consequently, the use of edible flowers appears to be beneficial in the prevention and supportive treatment of chronic diseases, including diabetes, cardiovascular diseases, as well as various types of cancer [26]; however, studies on their composition and/or health-promoting properties often focus on fresh flowers of the most popular species. 

The growing body of knowledge on the chemical composition and biological properties of edible flowers makes them attractive in the development of functional foods e.g., bars for athletes [27]; they meet dietary requirements as low-calorie food, as well as being vegetarian and vegan, and moreover, the visual appeal, due to a variety of colours and shapes, and the health-promoting properties of edible flowers fit well with the current trend promoting natural and healthy foods. Despite many studies on the composition and properties of edible flowers, there is still a lack of comparative data on their protein content or analyses of the different fractions of dietary fibre in freeze-dried flowers, which are gaining popularity in the food market.

In view of the above, the aim of this study was to determine the content of dietary fibre (total fibre and its soluble and insoluble fractions) and total protein in selected edible flowers. For the first time, the scope of the study includes a comparison of herbaceous and woody plants (trees and shrubs), and the *Oleaceae* and *Asteraceae* families.

## 2. Materials and Methods

Flower petals (*Magnolia × soulangeana*, *Sambucus nigra* L., *Syringa vulgaris* L. (violet), *Syringa vulgaris* L. (white), *Robinia pseudoacacia*, *Forsythia × intermedia*, *Cichorium intybus* L., *Bellis perennis*, *Tussilago farfara* L., *Calendula officinalis* L., *Taraxacum officinale* F.H. Wiggers coll., *Centaurea cyanus* L.) gathered in flowering from green areas (53°26′17″ N, 14°32′32″ E) served as the study material for this research (Table 1). Flowers of both cultivated and wild species were used in this study.

*Magnolia × soulangeana*, *Sambucus nigra* L., *Syringa vulgaris* L. (violet), *Syringa vulgaris* L. (white), *Robinia pseudoacacia, Forsythia × intermedia, Cichorium intybus* L., *Bellis perennis, Tussilago farfara* L., *Taraxacum officinale* F.H. Wiggers coll., *Centaurea cyanus* L. came from suburban green areas (parks, forests, wastelands) of the city of Szczecin (West Pomeranian Province, Poland). The species *Calendula officinalis* L. was sown and cultivated in home gardens in the suburbs of the city of Szczecin. The harvested plant (0.5 kg each flower) was assessed by a botanist. In species of the family Asteraceae, lingual flowers were used for the study. The collected flowers underwent lyophilisation in a lyophilisator (0.735 mmHg/−20 °C; Alpha 1-2 LD plus) and were then subjected to homogenisation by grinding to a powder in a food homogeniser (FOSS 2094).

The protein, and fibre contents were determined according to the method described by AOAC [28]. Total, insoluble and soluble dietary fibre was determined according to the enzymatic-gravimetric method; the K-TDFR 01/05 procedure (Megazyme International, Wicklow, Ireland) was conducted using Fibertec 1023 (FOSS, Hilleroed, Denmark).

Crude protein (N × 6.25) was measured by the Kjeldahl method, in a Büchi Distillation Unit B−324 (Büchi Labortechnik AG, Flawil, Switzerland). The Kjeldahl method was used as described by AOAC [28,29]. 

In all the experiments, three samples were analysed, and all the assays were carried out at least in triplicate. The statistical analysis was performed using Stat Soft Statistica 13.0 and Microsoft Excel 2017. The results are expressed as mean values and standard deviation (SD). Distributions of values for each parameter were analysed using the Shapiro–Wilk test. In order to assess the differences between examined parameters, the Kruskal–Wallis test was used. The Spearman test was used to calculate the correlation coefficient. Differences were considered significant at *p* ≤ 0.05. 

## 3. Results

Edible flowers are characterised by a high content of dietary fibre, especially its insoluble fraction. The total fibre (TF) content in the studied material ranged from 13.22 to 62.33 g/100 g. The highest content (62.33 g/100 g) was found in the petals of marigold, and the lowest (13.22 g/100 g) in magnolia. The content of insoluble dietary fibre (IDF) ranged from 8.69 (magnolia) to 57.54 g/100 g (marigold). The lowest content of soluble dietary fibre (SDF) was observed in white lilac petals (1.35 g/100 g), and the highest in cornflower petals (7.46 g/100 g) (Table 2). Statistically significant differences in terms of this parameter were noted in *Magnolia* × *soulangeana*, *Syringa vulgaris* L., *Forsythia* × *intermedia, Bellis perennis*, *Calendula officinalis* and *Centaurea cyanus* L. (*p* ≤ 0.05) (Table 2). 

Flowers are also a good, though underappreciated, source of plant protein, with content ranging from 8.70 g/100 g to 21.61 g/100 g of freeze-dried flowers. The lowest protein content was found in marigold flowers, and the highest in magnolia (Table 2). 

A very strong negative correlation was observed between protein content and that of total fibre (−0.7661; *p* ≤ 0.05) and insoluble fibre (−0.8217; *p* ≤ 0.05). In other words, flowers which are a good source of total protein contain significantly less total dietary fibre.

A statistically significant positive correlation between the different fractions of dietary fibre was also found. A positive correlation was observed between total fibre and insoluble fibre (0.9801; *p* ≤ 0.05), as well as soluble fibre (0.5826; *p* ≤ 0.05). A moderate correlation in the Spearman test was also noted between the insoluble and soluble fractions (0.4791; *p* ≤ 0.05). 

The content of the studied parameters differed between *Oleaceae* and *Asteraceae*. Flowers of species belonging to *Asteraceae* were observed to have a significantly higher content of total fibre and its insoluble and soluble fractions (Table 3). On the other hand, flowers from the olive family were characterised by a higher total protein content; thus, nutritional content is species-specific.

Statistically significant differences were noted in all parameters between herbaceous and woody plants (*p* ≤ 0.05). Herbaceous plants had higher levels of total and insoluble fibre, while woody plants were characterized by higher levels of soluble fibre and total protein (Table 4). It is worth adding that all herbaceous plants represented the *Asteraceae* family. 

95% confidence interval (CI) for mean and median values added as a Supplement (Table S1).

## 4. Discussion

Nowadays, edible flowers have become an extremely popular food ingredient. Flower petals not only make for unique and incredible food presentation, but also add an interesting flavour; while they are usually chosen for their appearance and/or taste, they also have health-promoting effects attributable to a wealth of various bioactive compounds—though this is unfortunately little known. Edible flowers are very rarely regarded as a source of valuable dietary fibre or protein, despite containing high levels of those nutrients, as shown in this study. The use of edible flowers, e.g., as an addition to bars or cocktails, which are a source of dietary fibre in the diet of athletes, seems to be an alternative and modern solution.

The present study showed that the average content of total fibre in freeze-dried flowers was 30.99 ± 14.41 g/100 g dry weight, with 26.64 ± 13.55 g/100 g of insoluble fibre, and as little as 5.97 ± 1.35 g/100 g of soluble fibre. The average protein content amounted to 14.24 ± 3.92 g/100 g. The highest content of total fibre and its insoluble fraction was found in flowers representing the *Asteraceae*: marigold, cornflower, chicory and daisy, popular herbaceous flowers. Flowers of the common daisy and cornflower, as well as elderflower and magnolia were characterised by a high content of soluble fibre. Interestingly, the content of soluble fibre in elderflower (5.97 g/100 g) is more than four times higher than that in lilac (1.35 g/100 g); these plants are often confused with each other, despite being members of different taxa.

The average daily requirement for dietary fibre is 25 g, also in the diet of athletes, and 100 g of freeze-dried marigold contains 62.33 g of total dietary fibre. One tablespoon (12 g) of freeze-dried calendula petals contains 7.44 g of fibre, that is, 30% of an adult’s recommended daily allowance (RDA) for this nutrient. Consuming 40 g a day, or about 3 tablespoons, will meet the daily standard. For the sake of comparison, the content of dietary fibre in wheat bran is 50 g/100 g, in wheat germ 15.5 g/100 g, and in flaxseed 27.3 g/100 g; it is therefore evident that edible flowers are as good a source of dietary fibre as some of the best known high-fibre foods.

The gut microflora can play a vital role in the health and performance of athletes [30]. The latest scientific research emphasises that high-protein diets of athletes, without sufficient fibre consumption, adversely affect the intestinal microflora; however, the positive effect of exercise on the gut microflora depends on the consumption of protein and dietary fibre. Increasing numbers of athletes will use probiotics. It is worth emphasising that in order to obtain a beneficial effect of the intervention, the diet should contain fibre, i.e., a prebiotic [30,31]. The latest randomised clinical trial confirms that the consumption of partially hydrolysed guar gum fibre as a prebiotic improves the gastrointestinal health of healthy athletes [32].

Furthermore, consumption of fibre-rich foods minimises the risk of developing a number of diseases. One of the leading causes of death in developed countries is cardiovascular disease. A strong correlation has been documented between the risk of cardiovascular disease and the consumption of high-fibre products. Several clinical trials have demonstrated that an increased content of whole-grain products in the diet significantly lowered blood cholesterol levels and improved the ratio of high-density lipoprotein (HDL) to low-density lipoprotein (LDL), as well as reducing the risk of a heart attack [33]. It has been reported that the risk of death from coronary heart disease was reduced by 17–35% with every additional 10 g of dietary fibre intake [34,35]. Dietary fibre was also found to lower the concentration of C-reactive protein (CRP), a marker of inflammation in the body and a predictor of coronary heart disease [36]. Increased intake of dietary fibre significantly improves overall intestinal health and reduces the risk of polyps and the incidence of cancer, notably colon and colorectal cancer; this has been confirmed in clinical trials and meta-analysis [7,37]. A higher consumption of dietary fibre is associated with shorter retention of intestinal contents, which in turn reduces the contact time between the cells of the intestinal mucosa and potentially carcinogenic substances. A reduced risk of disease may also be attributed to a higher intake of antioxidants, provided by fibre-rich foods [7]. High-fibre cereal products slow down the digestion of food, affecting postprandial glycemia and insulinemia; the phenomenon may also be due to delayed or decreased intestinal absorption [7]. A study involving 90,000 women and 45,000 men documented that a diet rich in whole-grain products reduces the risk of developing type 2 diabetes by 30% [33]. The effectiveness of increased fibre intake in this disease is also supported by meta-analyses [38,39,40]. It has been documented that dietary fibre intake is associated with a lower risk of metabolic syndrome (MetS) [39,40]. Increasing fibre consumption may effectively enhance satiety after a meal, which can help maintain a healthy weight or support weight loss [41]. Please note that the correlation between increased dietary fibre intake (by 8 g for every 1000 kcal) and weight loss was found to be independent of many other factors, including age and physical activity [42].

Dietary fibre can be categorized according to its source, solubility, fermentability, and physiological effects. Flowers rich in insoluble fibre (calendula, cornflower) may help ease constipation. Fibre will stimulate intestinal peristalsis, and absorb water, consequently increasing the volume of faeces and facilitating defecation. 

Cornflower and daisy (flowers with a high content of soluble dietary fibre) can be helpful for people who experience problems with satiety and maintaining a healthy body weight; this type of fibre can accelerate the rate of satiation. Soluble fibre clears out unwanted metabolic waste products and reduces cholesterol levels in the body. 

There are very few studies on the content of dietary fibre in edible flowers; moreover, those studies do not cover all the species included in this paper, and only quantify total dietary fibre without taking into account its fractions, which was done in our study for the first time. Our study is also the first to compare the *Oleaceae* and *Asteraceae* families, as well as woody vs. herbaceous plants.

In one study, eight plant species from Mexico were examined for total fibre content. The highest content was found in the coral tree *(Erythrina caribaea*), amounting to 17.7 g of total fibre per 100 g, followed by *Aloe vera*, with 13.8 g/100 g [43]. Rachkeeree et al. examined the fibre content in eight edible flowers of the ginger family (*Zingiberaceae*). The collected material was dried in an oven at 45 °C for 48 h and then ground to a fine powder. The plants included in the study are used in Thailand mainly for medicinal purposes, such as gastrointestinal ailments. The authors concluded that the studied flowers may be a good source of dietary fibre; however, their content averaged 0.58–3.58 g/100 g of the dried flowers [44]. In a study by the same research team, the fibre content in the flowers of *Bauhinia variegate* L., *Gmelina arborea* Roxb., *Shorea roxburghii* G. Don and *Viburnum* *inopinatum* Craib was found to be comparable to the present results. *Viburnum inopinatum* Craib had the highest content of dietary fibre (25.26 ± 1.23 g/100 g), while *Bauhinia variegate* L. had the highest protein content (10.26 g/100 g) [45]. The fibre content in other edible flowers, such as: *Tropaeolum majus* (monks cress)*, Tagetes erecta* (Mexican marigold)*,* and *Spilanthes olereacea* (paracress) amounted to 4.51 g, 9.20 g, and 10.11 g/100 g fresh weight, respectively [46]. The flowers of heather *(Calluna vulgaris* L.) were also found to be a noteworthy source of fibre. Its content in fresh flowers was 38.96 g/100 g. The authors of the study noted that the fibre content in heather is considerably higher when compared to green vegetables, such as chives, coriander, mint and parsley leaf [15]. De Bona et al. analysed, among other things, the fibre content in freeze-dried flowers of *Tropaeolum pentaphyllum*. The total fibre content was 5.22 g/100 g, insoluble fibre 3.34 g/100 g, and soluble fibre 1.88 g/100 g [47]. *Viola* × *wittrockiana* (pansy) and *Antirrhinum majus* (snapdragon) have also been tested for dietary fibre content. Pansy proved to be a better source of fibre, with a content of 5.09 g/100 g, while snapdragon contained 3.48 g/100 g of fresh material [20].

The presented results suggest that the majority of the studied flowers, including *T. pentaphyllum, Viola × wittrockiana, Antirrhinum majus,* are not as good a source of fibre as calendula or cornflower examined in this study. The differences may be due to the fact that those researchers examined fresh or traditionally air-dried flowers. The nutrient content is also determined by the species, family and form of the plant.

The flowers used in our study were first freeze-dried, which is one of the best food preservation methods available today. By removing water at a low temperature, chemical and enzymatic reactions can be inhibited, making it possible to obtain a safe and microbiologically clean product of a very high quality [48]. The freeze-drying process, therefore, extends the shelf life of the products. Compared to other food preservation methods, it maintains the nutritional value and sensory attributes of the products. Freeze-dried foods keep their fresh colour, taste and smell better than products obtained by other drying methods. The process also preserves valuable nutrients. During freeze-drying, vitamins and proteins, the most important nutrients, are not degraded [48]. Freeze-dried foods do not require any artificial food additives, such as preservatives, flavour enhancers or artificial flavours. The use of this technique seems to be the best technological solution for the preservation of flowers, not only because of preserving nutritional value and organoleptic characteristics, but also due to the sometimes very short flowering period. Freeze-drying is an excellent method for processing delicate material, such as petals of edible flowers, which can then be added in this form to various dishes to increase dietary fibre intake.

Edible flowers were found to be a rich source of protein, notably the flowers of magnolia, elderflower, black locust, and the common lilac with dark purple petals. The mean protein content was 14.24 ± 3.92 g/100 g raw material. For the sake of comparison, similar levels can be found in 100 g of tofu (14 g) and buckwheat groats (13 g). Meat-free diets, i.e., vegetarian and vegan, which exclude or significantly restrict animal protein, are extremely popular today. The search for a readily available, diverse source of protein, especially for people on such diets, seems essential. Edible flowers can contribute to the daily supply of protein, but further research is needed, especially to analyse the amino acid profile of different plant species.

It is also important to remember that increased amounts of dietary fibre should be incorporated in the diet gradually; this will reduce the possibility of adverse gastrointestinal symptoms such as abdominal pain, bloating, or increased gas. A high-fibre diet is contraindicated in a number of conditions, including gastrointestinal inflammation, inflammatory conditions of the pancreas, stomach, bile ducts and intestines, gastric and duodenal ulcers, as well as postoperative conditions. It should be emphasised that the fibre fraction and its amount should be selected according to the sport discipline, as well as the time, for example, to take part in competitions, in order to fully use its pro-health potential, in particular, the impact on the intestinal microflora.

## 5. Conclusions

Today, both consumers and manufacturers are paying increasing attention to the quality of foods and their content of individual nutrients. It turns out that the much-underrated edible flowers can be an excellent source of fibre and protein.

Educating the public and promoting edible flowers can contribute significantly to their incorporation into the daily diet on a wider scale. Apart from enhancing the taste and appearance of food, it will, most importantly, improve health. Increased fibre intake may reduce the risk of cardiovascular disease, including heart attack, and lower blood cholesterol levels; it will also have a positive effect on postprandial blood glucose levels, and in this way aid in the treatment and prevention of diabetes. Incorporating dried edible flowers in meals (about 10–20 g, i.e., one or two tablespoons) may be helpful in the prevention of colorectal cancer, by reducing gut retention time, and the contact time of toxins with the intestinal mucosa; it is also worth remembering that fibre effectively contributes to the satiety effect after meals, which will be helpful in the treatment or prevention of obesity.

Freeze-dried edible flowers, especially calendula and cornflower from the *Asteraceae* family of herbaceous plants, can be a valuable source of natural fibre in the daily diet. The flowers of woody plants, notably those from the *Oleaceae* family, were found to be a valuable source of protein. Because of their versatile nature, edible flowers can be used as functional food for athletes.

## Figures and Tables

**Table 1:** Characteristics of edible flowers.

Species	Familly	Form	Common Name	Colour of Petals	Flowering Time
*Magnolia* × *soulangeana*	*Magnoliaceae*	woody	saucer magnolia	white, rose	April–May
*Sambucus nigra* L.	*Adoxaceae*	woody	elderberry, black elder	white	May–June
*Syringa vulgaris* L. (violet)	*Oleaceae*	woody	lilac, common lilac	violet	May–June
*Syringa vulgaris* L. (white)	*Oleaceae*	woody	lilac, common lilac	white	May–June
*Robinia pseudoacacia*	*Fabaceae*	woody	black locust	white	May–June
*Forsythia* × *intermedia*	*Oleaceae*	woody	border forsythia	yellow	March–April
*Cichorium intybus* L.	*Asteraceae*	herbaceous	common chicory	blue	May–September
*Bellis perennis*	*Asteraceae*	herbaceous	daisy	white	March–November
*Tussilago farfara* L.	*Asteraceae*	herbaceous	coltsfoot	yellow	March–May
*Calendula officinalis* L.	*Asteraceae*	herbaceous	pot marigold, common marigold, ruddles or Scotch marigold	orange	June–September
*Taraxacum officinale* F.H. *Wiggers coll.*	*Asteraceae*	herbaceous	dandelion or common dandelion	yellow	April–August
*Centaurea cyanus* L.	*Asteraceae*	herbaceous	cornflower or bachelor’s button	blue	July–August

**Table 2:** The content of dietary fibre (total, soluble and insoluble fractions) and total protein in edible flowers.

	TF (Total Fibre) g/100 g	IDF (Insoluble) g/100 g	SDF (Soluble) g/100 g	Protein g/100 g
Systematic Name	Mean ± SD	Mean ± SD	Mean ± SD	Mean ± SD
*Magnolia* × *soulangeana* ^a^	13.22 ± 0.94 ^g,h,j,l^	8.69 ± 1.71 ^g,h,j,l^	4.53 ± 0.77	21.61 ± 0.04 ^d,g,h,j,l^
*Sambucus nigra* L. ^b^	29.13 ± 1.31	23.16 ± 1.88	5.97 ± 0.57 ^c,d,f^	19.70 ± 0.02 ^h,j,l^
*Syringa vulgaris* L. (violet) ^c^	17.21 ± 0.70 ^h,j,l^	13.96 ± 0.49 ^g,h,j,l^	3.24 ± 0.21 ^b,h,l^	15.63 ± 0.12 ^j,l^
*Syringa vulgaris* L. (white) ^d^	25.89 ± 0.75	24.54 ± 0.82	1.35 ± 0.07 ^b,h,l^	12.41 ± 0.07 ^a^
*Robinia pseudoacacia* ^e^	28.17 ± 4.20	24.32 ± 3.71	3.85 ± 0.49	17.83 ± 0.01 ^h,j,l^
*Forsythia* × *intermedia* ^f^	18.47 ± 1.34 ^h,j,l^	16.02 ± 1.70 ^h,j,l^	2.45 ± 0.36 ^b,h,l^	14.51 ± 0.01
*Cichorium intybus* L. ^g^	34.23 ± 0.04 ^g^	30.05 ± 0.38 ^a,c^	4.18 ± 0.42	12.45 ± 0.48 ^a^
*Bellis perennis* ^h^	38.25 ± 0.00 ^a,c,f^	32.12 ± 0.64 ^a,c,f^	6.13 ± 0.64 ^c,d,f^	11.10 ± 0.28 ^a,b,e^
*Tussilago farfara* L. ^i^	24.91 ± 0.90 ^j^	20.82 ± 0.84 ^j,l^	4.25 ± 0.06	14.08 ± 0.21
*Calendula officinalis* L. ^j^	62.33 ± 9.17 ^a,c,f,i^	57.54 ± 8.32 ^a,c,f,i^	4.79 ± 0.86	8.70 ± 0.01 ^a,b,c,e^
*Taraxacum officinale* F.H. Wiggers coll. ^k^	26.97 ± 0.86	22.87 ± 0.73	4.10 ± 0.13	13.24 ± 0.30
*Centaurea cyanus* L. ^l^	53.06 ± 0.62 ^a,c,f^	45.57 ± 0.77 ^a,c,f,i^	7.46 ± 0.15 ^c,d,f^	9.58 ± 0.11 ^a,b,c,e^

Data represent the mean values ± standard deviations of the three technical replicates. Different letters (a–l) in superscripts represent statistically significant differences in nutrients (*p* ≤ 0.05).

**Table 3:** The content of dietary fibre (total, soluble and insoluble fractions) and total protein in *Oleaceae* and *Asteraceae* family.

	TF (Total Fibre) g/100 g	IDF (Insoluble) g/100 g	SDF (Soluble) g/100 g	Protein g/100 g
Family	Mean ± SD	Mean ± SD	Mean ± SD	Mean ± SD
Oleaceae *	20.52 ± 3.89 ^&^	18.18 ± 2.35 ^&^	4.65 ± 0.79 ^&^	14.18 ± 1.38 ^&^
*Asteraceae* ^&^	39.97 ± 14.01 *	34.81 ± 13.35 *	5.16 ± 1.32 *	11.53 ± 1.99 *

Data represent the mean values ± standard deviations of the three technical replicates. * and ^&^ in superscripts represent statistically significant differences in nutrients (*p* ≤ 0.05). *Oleaceae* * family (*n* = 3): *Syringa vulgaris* L. (violet, white), *Forsythia × intermedia;* *Asteraceae* ^&^ family (*n* = 6) *Cichorium intybus* L., *Bellis perennis, Tussilago farfara* L., *Calendula officinalis* L., *Taraxacum officinale* F.H. Wiggers coll., *Centaurea cyanus* L.

**Table 4:** The content of dietary fibre (total, soluble and insoluble fractions) and total protein in *woody* and *herbaceous plant*.

	TF (Total Fibre) g/100 g	IDF (Insoluble) g/100 g	SDF (Soluble) g/100 g	Protein g/100 g
Form	Mean ± SD	Mean ± SD	Mean ± SD	Mean ± SD
Woody ^$^	22.02 ± 6.25 *^#^	18.45 ± 6.24 ^#^	3.57 ± 1.53 ^#^	16.95 ± 3.17 ^#^
Herbaceous ^#^	39.97 ± 14.01 ^$^	34.81 ± 13.35 ^$^	5.16 ± 1.32 ^$^	11.53 ± 1.99 ^$^

Data represent the mean values ± standard deviations of the three technical replicates. ^$^ and ^#^ in superscripts represent statistically significant differences in nutrients (*p* ≤ 0.05). Woody plants ^$^ (*n* = 6): *Magnolia × soulangeana, Sambucus nigra* L., *Syringa vulgaris* L. (violet), *Syringa vulgaris* L. (white), *Robinia pseudoacacia, Forsythia × intermedia*; herbaceous plants * (*n* = 6): *Cichorium intybus* L., *Bellis perennis, Tussilago farfara* L., *Calendula officinalis* L., *Taraxacum officinale* F.H. *Wiggers coll., Centaurea cyanus* L.

## Data Availability

Not applicable.

## Supplementary Materials

The following supporting information can be downloaded at: https://www.mdpi.com/article/10.3390/nu14122470/s1, Table S1. Confidence intervals (CI) for mean and median values for protein and fiber content in edible flowers.

## References

[1] Karpecka E., Fraczek B. (2022). Macronutrients and Water–Do They Matter in the Context of Cognitive Performance in Athletes?. Balt. J. Health Phys. Act..

[2] Frączek B., Gacek M., Pięta A., Tyrała F., Mazur-Kurach P., Karpęcka E. (2020). Dietary Mistakes of Polish Athletes in Relation to the Frequency of Consuming Foods Recommended in the Swiss Food Pyramid for Active People. Rocz. Panstw. Zakl. Hig..

[3] Fraczek B., Warzecha M., Tyrala F., Pieta A. (2016). Prevalence of the Use of Effective Ergogenic Aids among Professional Athletes. Rocz. Państw. Zakładu Hig..

[4] Anderson J.W., Baird P., Davis R.H., Ferreri S., Knudtson M., Koraym A., Waters V., Williams C.L. (2009). Health Benefits of Dietary Fiber. Nutr. Rev..

[5] Raninen K., Lappi J., Mykkänen H., Poutanen K. (2011). Dietary Fiber Type Reflects Physiological Functionality: Comparison of Grain Fiber, Inulin, and Polydextrose. Nutr. Rev..

[6] Gulati A.S., Dubinsky M.C. (2009). Probiotics in Pediatric Inflammatory Bowel Diseases. Curr. Gastroenterol. Rep..

[7] Lattimer J.M., Haub M.D. (2010). Effects of Dietary Fiber and Its Components on Metabolic Health. Nutrients.

[8] Capuano E. (2017). The Behavior of Dietary Fiber in the Gastrointestinal Tract Determines Its Physiological Effect. Crit. Rev. Food Sci. Nutr..

[9] So D., Whelan K., Rossi M., Morrison M., Holtmann G., Kelly J.T., Shanahan E.R., Staudacher H.M., Campbell K.L. (2018). Dietary Fiber Intervention on Gut Microbiota Composition in Healthy Adults: A Systematic Review and Meta-Analysis. Am. J. Clin. Nutr..

[10] Becker W. (2005). European Nutrition and Health Report 2004. Scand. J. Nutr..

[11] Melin A., Tornberg Å.B., Skouby S., Møller S.S., Faber J., Sundgot-Borgen J., Sjödin A. (2016). Low-Energy Density and High Fiber Intake Are Dietary Concerns in Female Endurance Athletes. Scand. J. Med. Sci. Sports.

[12] Vitale K., Hueglin S. (2021). Update on Vegetarian and Vegan Athletes: A Review. J. Phys. Fit. Sports Med..

[13] Wirnitzer K., Boldt P., Lechleitner C., Wirnitzer G., Leitzmann C., Rosemann T., Knechtle B. (2019). Health Status of Female and Male Vegetarian and Vegan Endurance Runners Compared to Omnivores—Results from the NURMI Study (Step 2). Nutrients.

[14] Rop O., Mlcek J., Jurikova T., Neugebauerova J., Vabkova J. (2012). Edible Flowers—A New Promising Source of Mineral Elements in Human Nutrition. Molecules.

[15] Rodrigues H., Cielo D.P., Goméz-Corona C., Silveira A.a.S., Marchesan T.A., Galmarini M.V., Richards N.S.P.S. (2017). Eating Flowers? Exploring Attitudes and Consumers’ Representation of Edible Flowers. Food Res. Int. Ott. Ont.

[16] Chen N.-H., Sherrie W. (2017). Factors Influencing Consumers’ Attitudes towards the Consumption of Edible Flower. Food Qual. Prefer..

[17] Fernandes L., Casal S., Pereira J.A., Pereira E.L., Saraiva J.A., Ramalhosa E. (2019). Physicochemical, Antioxidant and Microbial Properties of Crystallized Pansies (Viola × Wittrockiana) during Storage. Food Sci. Technol. Int..

[18] Pires T.C.S.P., Dias M.I., Barros L., Calhelha R.C., Alves M.J., Oliveira M.B.P.P., Santos-Buelga C., Ferreira I.C.F.R. (2018). Edible Flowers as Sources of Phenolic Compounds with Bioactive Potential. Food Res. Int. Ott. Ont.

[19] Hazli U.H.A.M., Abdul-Aziz A., Mat-Junit S., Chee C., Kong K. (2019). Solid-Liquid Extraction of Bioactive Compounds with Antioxidant Potential from Alternanthera Sesillis (Red) and Identification of the Polyphenols Using UHPLC-QqQ-MS/MS. Food Res. Int..

[20] González-Barrio R., Periago M.J., Luna-Recio C., Garcia-Alonso F.J., Navarro-González I. (2018). Chemical Composition of the Edible Flowers, Pansy (Viola Wittrockiana) and Snapdragon (Antirrhinum Majus) as New Sources of Bioactive Compounds. Food Chem..

[21] Meurer M.C., Mees M., Mariano L.N.B., Boeing T., Somensi L.B., Mariott M., da Silva R.d.C.M.V.d.A.F., Dos Santos A.C., Longo B., Santos França T.C. (2019). Hydroalcoholic Extract of Tagetes Erecta L. Flowers, Rich in the Carotenoid Lutein, Attenuates Inflammatory Cytokine Secretion and Improves the Oxidative Stress in an Animal Model of Ulcerative Colitis. Nutr. Res. N. Y. N.

[22] Nowicka P., Wojdyło A. (2019). Anti-Hyperglycemic and Anticholinergic Effects of Natural Antioxidant Contents in Edible Flowers. Antioxidants.

[23] Yang P.-F., Yang Y.-N., Feng Z.-M., Jiang J.-S., Zhang P.-C. (2019). Six New Compounds from the Flowers of Chrysanthemum Morifolium and Their Biological Activities. Bioorganic Chem..

[24] Nguyen C., Baskaran K., Pupulin A., Ruvinov I., Zaitoon O., Grewal S., Scaria B., Mehaidli A., Vegh C., Pandey S. (2019). Hibiscus Flower Extract Selectively Induces Apoptosis in Breast Cancer Cells and Positively Interacts with Common Chemotherapeutics. BMC Complement. Altern. Med..

[25] Mlček J., Rop O. (2011). Fresh edible flowers of ornamental plants–A new source of nutraceutical foods. Trends Food Sci. Technol..

[26] Takahashi J.A., Rezende F.A.G.G., Moura M.A.F., Dominguete L.C.B., Sande D. (2020). Edible Flowers: Bioactive Profile and Its Potential to Be Used in Food Development. Food Res. Int..

[27] Gostin A., Waisundara V. (2019). Edible Flowers as Functional Food: A Review on Artichoke (Cynara Cardunculus L.). Trends Food Sci. Technol..

[28] Baur F.J., Ensminger L.G. (1977). The Association of Official Analytical Chemists (AOAC). J. Am. Oil Chem. Soc..

[29] Barbano D.M., Clark J.L., Dunham C.E., Flemin R.J. (1990). Kjeldahl Method for Determination of Total Nitrogen Content of Milk: Collaborative Study. J. AOAC Int..

[30] Hughes R.L., Holscher H.D. (2021). Fueling Gut Microbes: A Review of the Interaction between Diet, Exercise, and the Gut Microbiota in Athletes. Adv. Nutr..

[31] Son J., Jang L.-G., Kim B.-Y., Lee S., Park H. (2020). The Effect of Athletes’ Probiotic Intake May Depend on Protein and Dietary Fiber Intake. Nutrients.

[32] Kapoor M.P., Koido M., Kawaguchi M., Timm D., Ozeki M., Yamada M., Mitsuya T., Okubo T. (2020). Lifestyle Related Changes with Partially Hydrolyzed Guar Gum Dietary Fiber in Healthy Athlete Individuals – A Randomized, Double-Blind, Crossover, Placebo-Controlled Gut Microbiome Clinical Study. J. Funct. Foods.

[33] Slavin J.L., Jacobs D., Marquart L., Wiemer K. (2001). The Role of Whole Grains in Disease Prevention. J. Am. Diet. Assoc..

[34] Streppel M.T., Ocké M.C., Boshuizen H.C., Kok F.J., Kromhout D. (2008). Dietary Fiber Intake in Relation to Coronary Heart Disease and All-Cause Mortality over 40 y: The Zutphen Study. Am. J. Clin. Nutr..

[35] Pereira M.A., O’Reilly E., Augustsson K., Fraser G.E., Goldbourt U., Heitmann B.L., Hallmans G., Knekt P., Liu S., Pietinen P. (2004). Dietary Fiber and Risk of Coronary Heart Disease: A Pooled Analysis of Cohort Studies. Arch. Intern. Med..

[36] Ma Y., Griffith J.A., Chasan-Taber L., Olendzki B.C., Jackson E., Stanek E.J., Li W., Pagoto S.L., Hafner A.R., Ockene I.S. (2006). Association between Dietary Fiber and Serum C-Reactive Protein. Am. J. Clin. Nutr..

[37] Ma Y., Hu M., Zhou L., Ling S., Li Y., Kong B., Huang P. (2018). Dietary Fiber Intake and Risks of Proximal and Distal Colon Cancers. Medicine (Baltimore).

[38] Jovanovski E., Khayyat R., Zurbau A., Komishon A., Mazhar N., Sievenpiper J.L., Blanco Mejia S., Ho H.V.T., Li D., Jenkins A.L. (2019). Should Viscous Fiber Supplements Be Considered in Diabetes Control? Results From a Systematic Review and Meta-Analysis of Randomized Controlled Trials. Diabetes Care.

[39] Wei B., Liu Y., Lin X., Fang Y., Cui J., Wan J. (2018). Dietary Fiber Intake and Risk of Metabolic Syndrome: A Meta-Analysis of Observational Studies. Clin. Nutr..

[40] Chen J.-P., Chen G.-C., Wang X.-P., Qin L., Bai Y. (2018). Dietary Fiber and Metabolic Syndrome: A Meta-Analysis and Review of Related Mechanisms. Nutrients.

[41] Slavin J., Green H. (2007). Dietary Fibre and Satiety. Nutr. Bull..

[42] Tucker L.A., Thomas K.S. (2009). Increasing Total Fiber Intake Reduces Risk of Weight and Fat Gains in Women. J. Nutr..

[43] Sotelo A., López-García S., Basurto-Peña F. (2007). Content of Nutrient and Antinutrient in Edible Flowers of Wild Plants in Mexico. Plant Foods Hum. Nutr. Dordr. Neth..

[44] Rachkeeree A., Kantadoung K., Suksathan R., Puangpradab R., Page P.A., Sommano S.R. (2018). Nutritional Compositions and Phytochemical Properties of the Edible Flowers from Selected Zingiberaceae Found in Thailand. Front. Nutr..

[45] Suksathan R., Rachkeeree A., Puangpradab R., Kantadoung K., Sommano S.R. (2021). Phytochemical and Nutritional Compositions and Antioxidants Properties of Wild Edible Flowers as Sources of New Tea Formulations. NFS J..

[46] Navarro-González I., González-Barrio R., García-Valverde V., Bautista-Ortín A.B., Periago M.J. (2014). Nutritional Composition and Antioxidant Capacity in Edible Flowers: Characterisation of Phenolic Compounds by HPLC-DAD-ESI/MSn. Int. J. Mol. Sci..

[47] De Bona G.S., Boschetti W., Bortolin R.C., Vale M.G.R., Moreira J.C.F., de Rios A.O., Flôres S.H. (2017). Characterization of Dietary Constituents and Antioxidant Capacity of Tropaeolum Pentaphyllum Lam. J. Food Sci. Technol..

[48] Liu B., Zhou X. (2021). Freeze-Drying of Proteins. Methods Mol. Biol. Clifton NJ.

